# Stormwater influences phytoplankton assemblages within the diverse, but impacted Sydney Harbour estuary

**DOI:** 10.1371/journal.pone.0209857

**Published:** 2018-12-26

**Authors:** Deepa Varkey, Sophie Mazard, Thomas C. Jeffries, David J. Hughes, Justin Seymour, Ian T. Paulsen, Martin Ostrowski

**Affiliations:** 1 Department of Molecular Sciences, Macquarie University, Sydney, NSW, Australia; 2 School of Science and Health, Western Sydney University, Penrith, NSW, Australia; 3 University of Technology Sydney, Climate Change Cluster, Ultimo, NSW, Australia; Feroze Gandhi Degree College, INDIA

## Abstract

Sydney Harbour is subjected to persistent stress associated with anthropogenic activity and global climate change, but is particularly subjected to pulse stress events associated with stormwater input during episodic periods of high rainfall. Photosynthetic microbes underpin metazoan diversity within estuarine systems and are therefore important bioindicators of ecosystem health; yet how stormwater input affects their occurrence and distribution in Sydney Harbour remains poorly understood. We utilised molecular tools (16S/18S rRNA and *petB* genes) to examine how the phytoplankton community structure (both prokaryotes and eukaryotes) within Sydney Harbour varies between high and low rainfall periods. The relative proportion of phytoplankton sequences was more abundant during the high rainfall period, comprising mainly of diatoms, an important functional group supporting increased productivity within estuarine systems, together with cyanobacteria. Increased spatial variability in the phytoplankton community composition was observed, potentially driven by the steepened physico-chemical gradients associated with stormwater inflow. Conversely, during a low rainfall period, the proportion of planktonic photosynthetic microbes was significantly lower and the persistent phytoplankton were predominantly represented by chlorophyte and dinoflagellate sequences, with lower overall diversity. Differences in phytoplankton composition between the high and low rainfall periods were correlated with temperature, salinity, total nitrogen and silicate. These results suggest that increased frequency of high-rainfall events may change the composition, productivity and health of the estuary. Our study begins to populate the knowledge gap in the phytoplankton community structure and substantial changes associated with transient environmental perturbations, an essential step towards unravelling the dynamics of primary production in a highly urbanised estuarine ecosystem in response to climate change and other anthropogenic stressors.

## Introduction

Unicellular phytoplankton are significant contributors to primary production [1, 2], and biogeochemical cycling within estuaries [3]. Phytoplankton productivity is however strongly dependent on environmental conditions, such as the availability of light and nutrients, temperature and salinity [4]. Accordingly, phytoplankton assemblages are also highly dynamic and often characterised by species succession in response to environmental perturbations [5]. Alterations to phytoplankton assemblages have direct implications for the nutritional quality [2] and the number of trophic levels in the food chain [6–8], and thus may play important roles in biodiversity at higher trophic levels [9]. Phytoplankton may also be associated with negative impacts during blooms, often promoted by transient nutrient inputs, notably when eutrophication results in localised hypoxia and loss of benthic productivity which can both ultimately impact higher trophic species [10]. Therefore, examining phytoplankton assemblages and understanding how they respond to environmental perturbation is an important area of study and provides an insight into estuarine ecosystem health and functioning [11].

The last major assessment of phytoplankton assemblages in the Sydney Harbour estuary was undertaken some four decades ago [12]. Sydney Harbour is arguably the most biodiverse estuary in the world [13], supporting over 3000 species of metazoans due to high habitat heterogeneity [13–15]. As such the estuary holds great socio-economic and environmental importance for the surrounding population of Sydney, and Australia as a whole [16]. Yet, despite the ecological significance of Sydney Harbour, there is a paucity of taxonomic data on the phytoplankton community. Recent research on phytoplankton within the estuary has mainly centred on understanding the occurrence of harmful algal blooms [17, 18], rather than examining the primary producers *per se*. Therefore, phytoplankton diversity and the environmental conditions that shape the community in the estuary remain unexplored.

Furthermore, Sydney Harbour has been impacted by persistent stress associated with heavy industrialisation and urbanisation [19, 20] and more recently, by increases in sea surface temperature and the subsequent “tropicalization” of the harbour [21]. The estuary is also notably subjected to transient perturbations associated with stormwater inflow during periods of high rainfall, leading to elevated nutrient loading, higher suspended particulate matter and fluctuations in salinity [20, 22–25]. Unlike many other estuaries, there are no major freshwater rivers draining into the harbour, and thus it remains a well-mixed marine estuary for most of the year, with these infrequent, but large precipitation events acting as the major source of freshwater and nutrient loading [13, 23]. Recent efforts examining the benthic [26, 27] and bacterial communities [28], with specific focus on the implications for human health [29], identified the influence of transient environmental perturbations on these communities; yet, little attention has been paid to microbial primary producers.

Therefore, it remains unclear how transient perturbations during periods of high rainfall regulates overall phytoplankton diversity and how this affects primary production and thus ultimately, system biodiversity. This study intended to begin to fill that knowledge gap, focusing on how the phytoplankton community composition changes in response to episodic stormwater inflow within Sydney Harbour. This is particularly important at a time of increasing persistent stress associated with urbanisation and global warming, together with unknown implications for future rainfall patterns as a result of climate change.

## Materials and methods

### Sample collection

**Fig 1:**
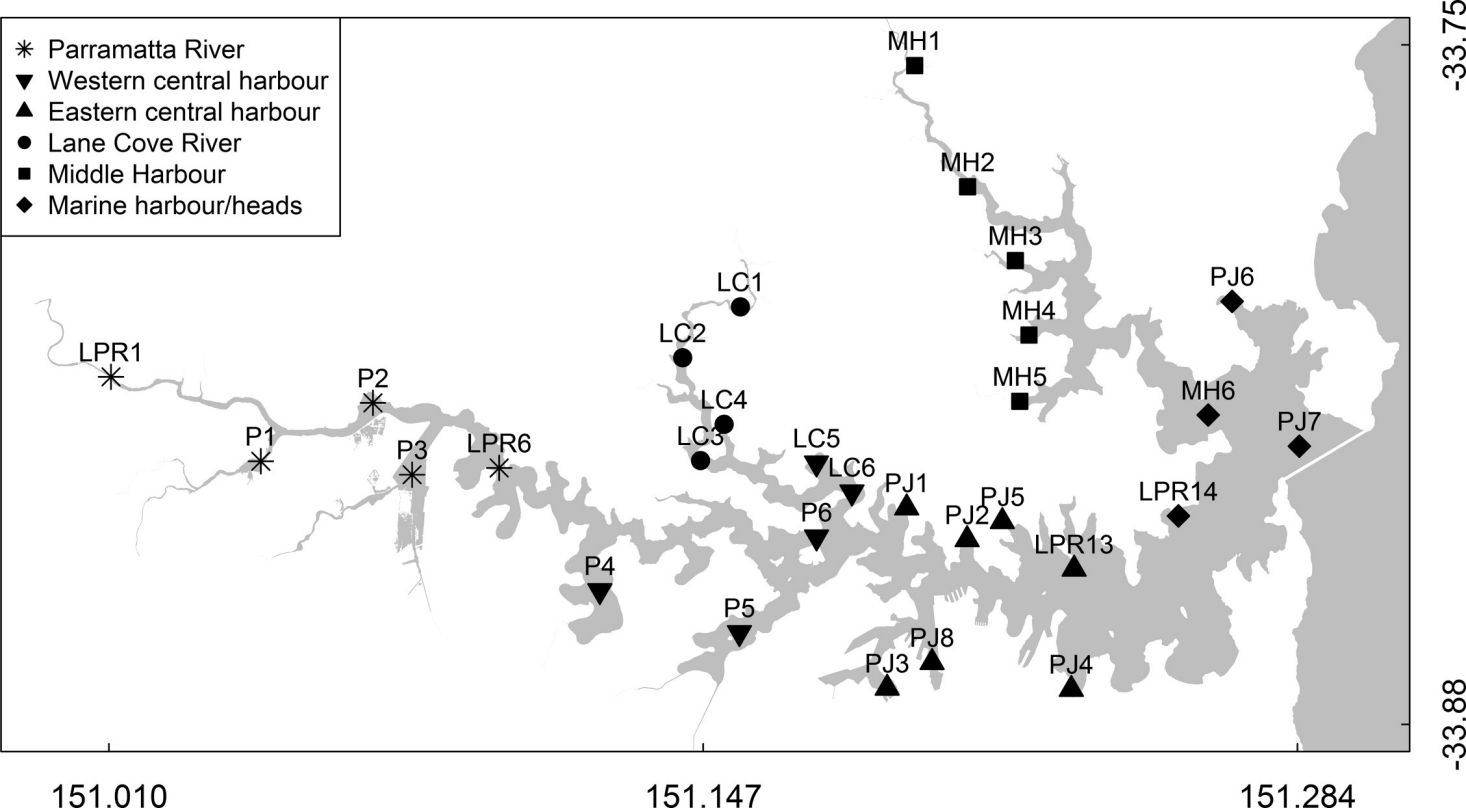
Map of the Sydney Harbour estuary depicting sampling sites. Symbols represent regions: Parramatta River (*), Western Central Harbour (▼), Eastern Central Harbour (▲), Lane Cove River (●), Middle Harbour (■) and Marine/Harbour Heads (♦).

Samples were collected from 30 sites in the Sydney Harbour estuary (30 km; as in [28]). Sample collection required no specific permissions since it was as part of a long term environmental monitoring program conducted by the Sydney Institute of Marine Science and did not involve any endangered or protected species. Sampling sites were grouped into six regions based on location: Parramatta River, Lane Cove River, Western Central Harbour, Eastern Central Harbour, Middle Harbour and Marine/Harbour Heads (Fig 1). Water conditions ranged from brackish in the uppermost reaches of the estuary to marine at mouth of the harbour. Sampling was conducted twice in 2013, once during a high rainfall period in February (late summer) and once during a prolonged low rainfall period in September (early spring). Water samples (2 l) from a depth of ~0.5 m at each site were filtered onto 0.2 μm polycarbonate membrane filters (Millipore, Australia) and stored at -20°C until further processing. Physico-chemical parameters were measured as part of [28]. Briefly, chlorophyll, O_2_, pH, temperature and turbidity were measured using a multi-parameter water quality probe (YSI-6600, Yellowstone Instruments, USA). Unfiltered sample (5 ml) was collected for total nitrogen (N) and total phosphorus (P) and for all other nutrient analyses, 80 ml was filtered using 0.45 μm cellulose acetate membrane syringe filter (Whatman). All nutrients were analysed on a LaChat 8500 using flow injection. Total N/total P and total dissolved N/total dissolved P were prepared using the modified alkaline peroxidisulfate autoclave digestion; nitrogen oxides, ammonium, phosphate (PO_4_) and silicate (Si) were analysed using standard methods; total suspended solids (TSS) were measured gravimetrically using standard method 2540 D (see references in [28] for details).

### Nucleic acid extraction

DNA was extracted from the polycarbonate membrane and Sterivex filters with a PowerWater DNA isolation kit (MO BIO, USA) using bead beating for cell lysis. DNA in nuclease-free water was stored at -80°C until used for amplification of phylogenetic markers. The concentration of DNA was measured using a NanoDrop 2000 UV spectrophotometer (Thermo Scientific, USA).

### Library preparation

Generation of amplicon libraries of the prokaryotic 16S rRNA gene was carried out with the universal eubacterial primers 926F (5’-AAACTYAAAKGAATTGACGG-3’) and 1392R (5’-ACGGGCGGTGTGTRC-3’). The preparation, processing and sequencing of these amplicons is described in [28].

To evaluate the eukaryotic community composition, the amplicon libraries of the eukaryotic 18S rRNA gene was generated with the universal V9 region primers (primer set 1380F and 1510R; [30]) attached with sequencing adaptors and indices (based on 16S rRNA gene metagenomic library preparation guide from Illumina, Inc.) for multiplex sequencing. Amplicon libraries for the 18S rRNA gene were prepared in 50 μl PCR reactions, each reaction contained 25 pmol each of forward and reverse Illumina primers, 0.8 mM dNTPs, 1X reaction buffer, 1 unit Taq polymerase (Qiagen, Australia) and 5–10 ng of template DNA. The PCR program comprised a denaturation step at 94°C for 5 min, followed by 30 cycles of denaturation at 94°C for 20s, annealing at 57°C for 20s, extension at 72°C for 30s, and a final extension step for 6 min at 72°C.

The high resolution phylogenetic marker, *petB* gene, which encodes the cytochrome *b*_*6*_ subunit of cytochrome *b*_*6*_*f* complex [31], was used to enhance the level of taxonomic resolution of the main prokaryotic phytoplankton group, the cyanobacterium *Synechococcus*. Specific primers for the *petB* gene, targeting the picocyanobacterial community [31], were attached with sequencing adaptors and indices that enabled multiplexed sequencing as per 16S rRNA gene metagenomic library preparation guide (Illumina Inc.). Amplicon libraries of the *petB* marker were prepared as done for the 18S rRNA gene with the following modifications: each reaction contained 20 pmol each of forward and reverse primers and 2.5 mM MgCl_2_ (Qiagen, Australia). The PCR program comprised a denaturation step at 95°C for 5 min, followed by 30 cycles of denaturation at 95°C for 30s, annealing at 55°C for 30s, extension at 72°C for 45s, and a final extension step for 6 min at 72°C.

Amplification products of *petB* and 18S rRNA were quantitated using Quant-iT PicoGreen dsDNA assay kit (Life Technologies, Australia). Amplicons from each site were pooled (15/10 ng DNA per site for 18S rRNA gene or *petB* respectively) and purified using Agencourt AMPure XP bead purification (Beckman Coulter, Inc., Australia) and eluted using nuclease-free water (Ambion, Australia). Purified multiplexed samples were sequenced on a 300bp Paired-End run (*petB*) or a 150bp Paired-End run (18S rRNA gene) using the Illumina MiSeq platform at Ramaciotti Centre for Genomics (NSW, Australia). Raw sequence files have been deposited at NCBI Sequence Read Archive (BioProject ID: PRJNA491799).

### Bioinformatic analyses

Microbial community structure data determined using universal 454 sequencing of 16S rRNA gene amplicons [28] was re-analysed using an in-house bioinformatic analysis pipeline based on the USEARCH64 program [32]. Briefly, the paired-end sequences for the 16S rRNA gene markers were joined using the FLASH algorithm [33]. Barcodes and primers were removed and all sequences were trimmed to 360 bp after quality filtering. Sequences with Ns and any less than 360 bp in length were discarded. *De novo* Operational Taxonomic Units (OTUs) were produced at 99% identity from the pool of dereplicated sequences after removal of singletons and chimeras. Taxonomy was assigned against the Silva 119 release 99% non-redundant reference database [34]. A mapping file and an OTU table were created by mapping the original file against the *de novo* OTUs using the python script uc2otutab.py. The relative abundance of sequences classified as cyanobacteria; chloroplasts, and cyanobacteria were extracted to estimate the relative proportion of phototrophic sequences at each site. Representative sequences classified as cyanobacteria were assigned to *Prochlorococcus* and *Synechococcus* sub clusters 5.1, 5.2 and 5.3 using a 16S rRNA gene reference phylogenetic tree from [31].

The paired-end 18S rRNA gene sequences were joined, cleaned and processed through the same USEARCH64-based pipeline as above with minor modifications. Briefly, sequences were processed through an initial dereplication, and then sorted into clusters at 97% identity. The taxonomy for each OTU was assigned against the Protist Ribosomal Reference (PR^2^) database [35] using Mothur classify.seqs command using Knn, numwanted = 3 [36].

The *petB* amplicon sequencing reads were processed using the same USEARCH64 pipeline as above with some modifications. Using QIIME [37] split_libraies.py, sequencing adaptors and primers were removed, sequences were trimmed to 280 bp and quality filtered. The cleaned sequences were dereplicated (using usearch–derep) and filtered to remove sequences with less than 4 representatives. *De novo* OTUs were produced by clustering dereplicated sequences at 97% identity, removing chimeras and further clustering at 94%. A mapping file was created containing *de novo petB* OTUs and *petB* closed reference sequences (using an up to date *petB* sequence database containing sequences from complete genomes, cloned bidirectional sequences from the Warwick and Roscoff culture collections, and bidirectional sequences from clone libraries of environmental amplicons [31, 38]. The quality trimmed reads were then searched against the mapping file to produce the OTU table. Representative sequences of *de novo* OTUs and the closed reference sequences were used to generate a multiple sequence alignment and a consensus phylogenetic tree in ARB [39] using Neighbour Joining and PhyML [40], which was then used to assign taxonomy and examine the phylogenetic placement of *de novo* OTUs.

### Data analyses

Statistical analysis was performed using R software v 3.4.2 [41] and Primer v6.0 [42]. For each phylogenetic marker, OTU counts per site were aggregated based on taxonomic assignment, total counts per site were rarefied to the lowest total count obtained (16S rRNA– 1130; 18S rRNA– 35232; *petB*– 1547) and scaled using square-root transformation. Sample sites were clustered based on Bray Curtis similarity of taxonomic abundance using hierarchical cluster analysis with SIMPROF test (p = 0.05). Analysis of similarity (ANOSIM) was performed to identify statistically significant clusters. Multidimensional scaling (MDS) plots were generated to visualise the separation of clusters based on taxonomic abundance. Similarity Percentage (SIMPER) analyses was performed to determine the contributions of taxonomic groups to the Bray Curtis dissimilarity between months and clusters. One-way analysis of variance (ANOVA) with TukeyHSD, after verifying normality, was used to test the statistical relationships between selected groups from the two sampling periods.

Environmental parameters were assessed using draftsman’s plots and log-transformed if distribution was skewed. Correlations between variables were explored and for those where the correlation coefficient was >0.8, one of the co-variables was removed. All retained variables were normalised for all subsequent analyses. RELATE and BEST (with Akaike information criterion) analyses with 999 permutations, were used to determine how well the resemblance matrices of environmental variables and taxonomic abundance matched, and which of the variables best explained patterns in taxonomic abundances. Distance-based linear modelling (DistLM) and distance-based redundancy analysis (dbRDA) were performed to examine taxonomic abundance variability explained by environmental variables.

## Results

### Physico-chemical conditions

The sampling period corresponded to a prolonged period of high rainfall (14.11 mm above the historical average) during the month sampled (February) [43]. Water temperature ranged from ~22–27.5°C, with higher temperatures corresponding to sites located within inland branches of the estuary (S1 Fig). Inland sites were characterised by lower salinities (as low as 12.26 PSU), due to their proximity to freshwater input sources. Salinity increased along the estuary, reaching 34 PSU towards the harbour entrance, thus consistent with fully oceanic conditions (S1 Fig). Average pH was 8.06 ±0.35 with the lowest values corresponding to the most inland sites in Lane Cove and Middle Harbour (S1 Fig). The average concentration of TSS was 7.2 ±5.3 mgL^-1^, which was lower towards the mouth of the harbour (S1 Fig). Nutrient (including oxidised, reduced and total N, PO_4_ and Si) concentrations were highest at the sites furthest inland, and considerably lower towards the harbour mouth (S2 Fig). Average dissolved O_2_ concentration was 9.18 ±2.4 mgL^-1^, highest in the Parramatta and Lane Cove Rivers, and the lowest was in site MH1 (2.4 mgL^-1^) in Middle Harbour.

During the low rainfall period (in September), rainfall was only half that of the typical monthly average. The range of measured temperatures was smaller (~17–20°C) and salinity was remarkably constant across the estuary (~34 PSU), except for the most inland sites of Parramatta and Lane Cove Rivers where it dropped to 28–30 PSU (S1 Fig). Furthermore, average pH (7.84 ±0.2) and TSS (3.5 ±3.7 mgL^-1^) were lower than during the high rainfall period. Excluding the most inland sites, average concentrations of oxidised and reduced N and PO_4_ were ~2-3x higher whilst average total N and Si amounts were only half that of the high rainfall period (S2 Fig). Generally, nutrient concentrations were higher in the inland branches, (i.e. closer to the stormwater input sources) compared to the main estuary (S2 Fig). Average dissolved O_2_ concentration was lower (8.6 ±0.8 mgL^-1^) and less variable across the estuary than during the high rainfall period.

### Temporal and spatial shifts in phytoplankton composition

The microbial community, including the prokaryotic, eukaryotic and cyanobacterial fractions, in the high rainfall period was significantly distinct from the community present during the low rainfall period in the Sydney Harbour estuary (ANOSIM: 16S rRNA—R statistic = 0.912; significance = 0.1%; 18S rRNA–R statistic = 0.52, significance = 0.1%; *petB*–R statistic = 1, significance = 0.1%).

All proportions of taxa mentioned below are as percentages of the total sequences for each phylogenetic marker (i.e. 16S rRNA, 18S rRNA or *petB* genes). Since eukaryotic phytoplankton can have a wide range of rRNA gene copy numbers (100s - 1000s; [44]), OTU relative abundances cannot be used as a direct quantitative measure of cell density [45]. However, OTU abundances are not completely independent of plankton numbers [45] therefore we have used it to compare the relative abundances of phytoplankton groups between sampling periods.

#### Microbial phototrophs: key differences between rainfall periods

**Fig 2:**
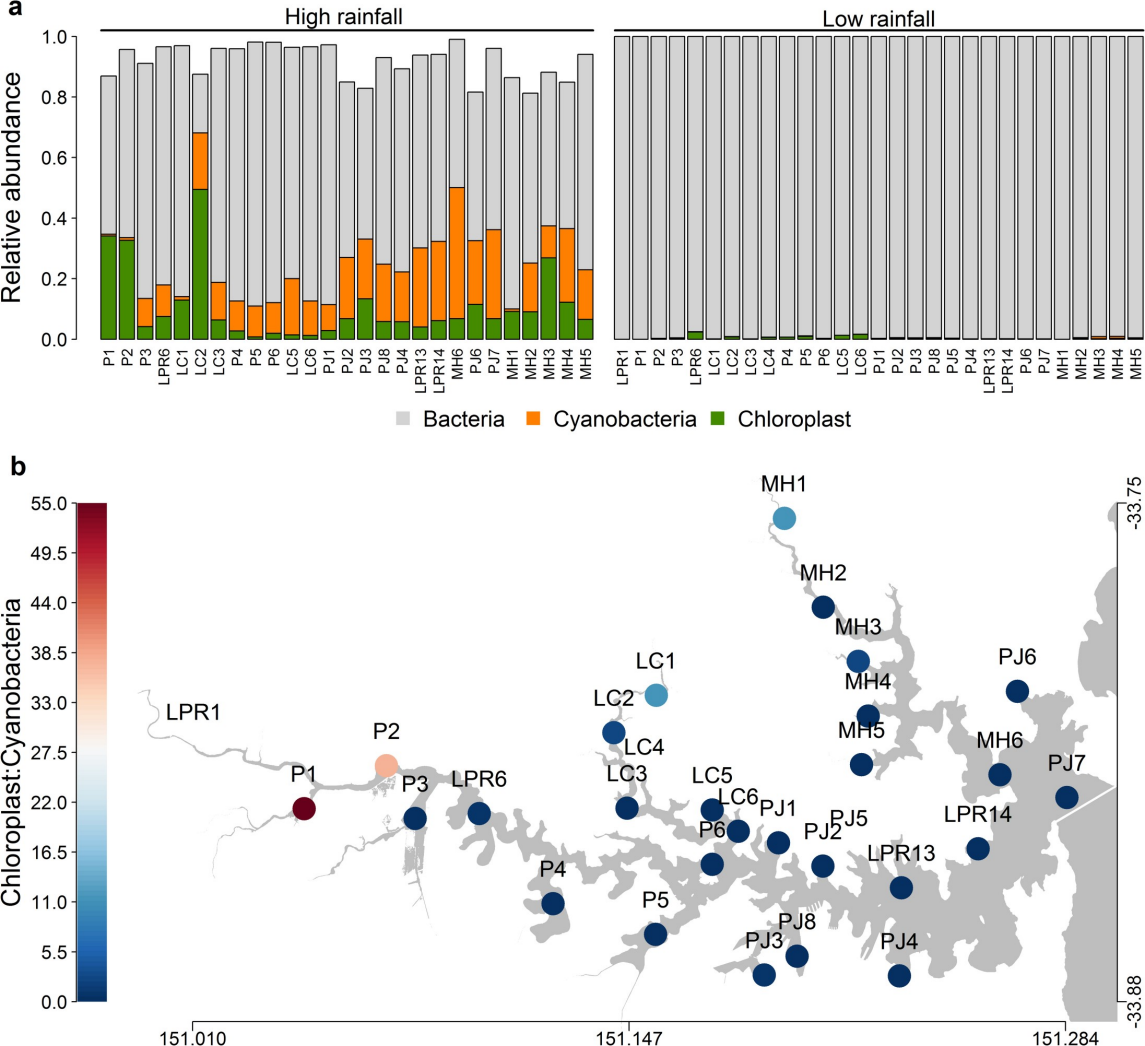
Relative proportions of microbial functional groups in the Sydney Harbour estuary, based on V6-V8 regions of 16S rRNA gene. **a)** Relative abundance of the heterotrophic (bacteria) and autotrophic (cyanobacteria and chloroplast) fractions, at each sampling site, under high and low rainfall conditions. Reads that did not fall in these functional categories have been excluded. **b)** Spatial representation of the ratio of eukaryotic (chloroplast) to prokaryotic (cyanobacteria) phytoplankton during high rainfall.

In the high rainfall period, the distinct microbial community (based on 16S rRNA gene; S3 Fig) was characterised by significantly higher relative abundance of the phototrophic components, chloroplast (13.6%) and cyanobacteria (15.3%), which were both less than 0.5% during low rainfall (p <0.05; Fig 2A). This pattern is consistent with the significantly higher concentrations of chlorophyll (11.88 μgL^-1^) during the high rainfall period relative to the low rainfall period (7.24 μgL^-1^; p <0.05; S1 Fig).

Spatial variability was evident during the high rainfall period with higher proportions of chloroplast than cyanobacterial 16S rRNA sequences in the most inland sites, i.e. P1 and P2 (Parramatta River), LC1 and LC2 (Lane Cove River) and MH1 and MH3 (Middle Harbour) [Fig 2B]. The rest of the estuary, particularly those at the mouth of the harbour, had an equal or higher percentage of cyanobacteria (Fig 2B). A major proportion of cyanobacteria was marine *Synechococcus*, mainly comprising (53–70% of *petB* sequences) the clade II lineage in the high rainfall period and clade I during low rainfall (S4 Fig).

#### Eukaryotic phytoplankton: Key differences between rainfall periods

**Fig 3:**
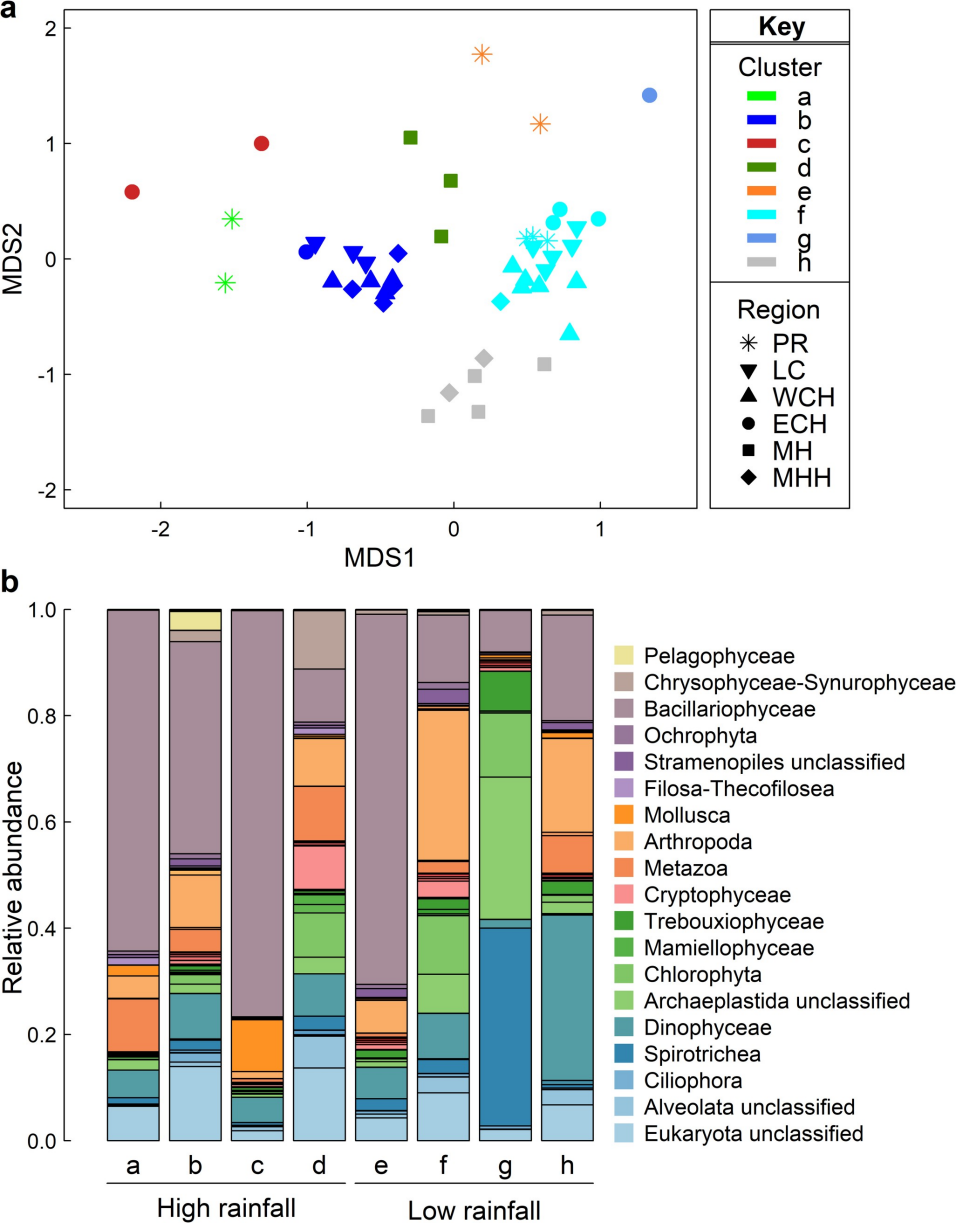
Eukaryotic community profile, based on 18S rRNA gene V9 region, in Sydney Harbour estuary during high and low rainfall periods. **a)** Multi-dimensional scaling plot of the community composition during the two periods. Samples are colour-coded based on clusters (assigned using hierarchical cluster analysis with SIMPROF test based on Bray-Curtis similarity) from the high rainfall period: **a** (light green), **b** (dark blue), **c** (red), **d** (dark green); and from the low rainfall period: **e** (orange), **f** (light blue), **g** (blue), **h** (grey). Symbols represent geographic region sampled: Parramatta River (*), Lane Cove River (●), Western Central Harbour (▼), Eastern Central Harbour (▲), Middle Harbour (■) and Marine/Harbour Heads (♦). **b)** Relative abundance profile of the eukaryotic community during the sampling periods, at taxonomic level of family or lower. Each bar represents the cluster of sites, as above. The rarefied number of reads assigned to each lineage was averaged across sites of each cluster.

During the high rainfall period, the distinct eukaryotic community (based on 18S rRNA gene; Fig 3a) was dominated by Bacillariophyceae (diatoms), in particular polar-centric and unclassified Bacillariophyceae, which occurred in an average relative abundance of 42% across the estuary (Fig 3B; S5 Fig). During the low rainfall period, diatoms had notably lower relative abundance (18% on average across the estuary) with pennate species most prevalent whilst 18S rRNA OTUs assigned to Dinophyceae (13.1%) and Archaeplastida sub-groups (17.9%), were almost twice that of the high rainfall period (Fig 3B; S5 Fig).

During the high rainfall period, sites close to potential stormwater point sources in Lane Cove (cluster c) and Parramatta (cluster a) Rivers had higher relative abundance of diatoms than the main estuary (Fig 3), each region distinguished by specific diatom taxa (S5 Fig). During the low rainfall period, only the most inland sites were distinct from the other sites in the estuary whereby LPR1 and P1 (cluster e) of Parramatta River were characterised by high diatom prevalence whilst LC1 (cluster g) of Lane Cove River had the lowest relative abundance of diatoms and the highest proportion of the ciliate, *Spirotrichea* (Fig 3). The Middle Harbour community was differentiated from the other sites in the estuary during both sampling periods with twice the proportion of OTUs assigned to Archaeplastida, Chrysophyceae-Synurophyceae and Cryptophyceae in the high rainfall period (cluster d) and the highest relative abundance of Dinophyceae (dinoflagellate) OTUs during low rainfall (cluster h; Fig 3).

### Influence of environmental variables on phytoplankton composition

**Fig 4:**
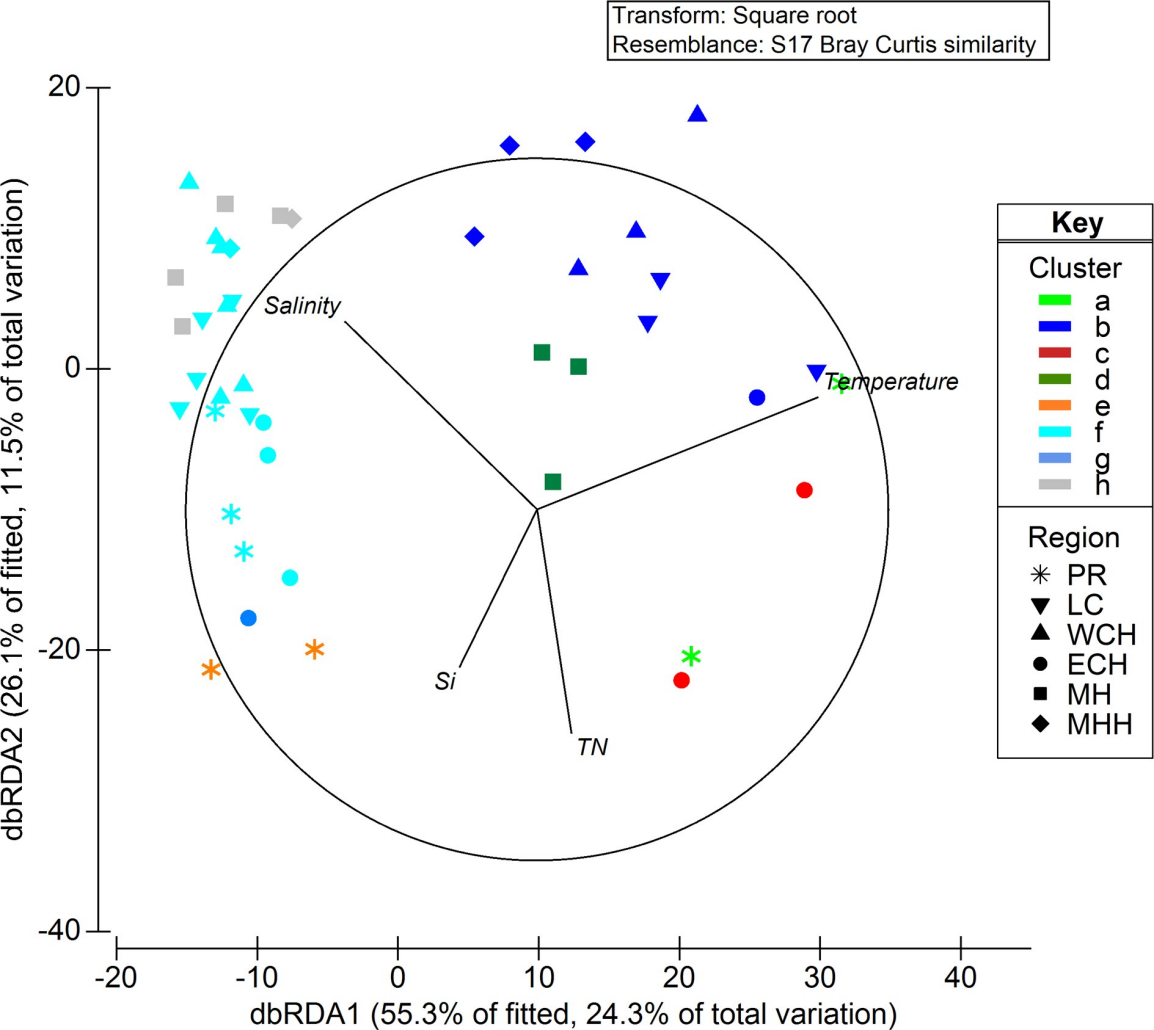
Distance-based redundancy analysis plot of the eukaryotic community structure (using 18S rRNA gene V9 region) for the Sydney Harbour estuary, during high and low rainfall periods. Samples are colour-coded based on clusters (assigned using hierarchical cluster analysis with SIMPROF test based on Bray-Curtis similarity) for the high rainfall period: **a** (light green), **b** (dark blue), **c** (red), **d** (dark green); and for the low rainfall period: **e** (orange), **f** (light blue), **g** (blue), **h** (grey). Symbols represent geographic region sampled: Parramatta River (*), Lane Cove River (●), Western Central Harbour (▼), Eastern Central Harbour (▲), Middle Harbour (■) and Marine/Harbour Heads (♦). TN–Total nitrogen; Si–Silicate.

Patterns in the eukaryotic taxonomic composition significantly correlated with the variation of environmental variables (R statistic = 0.66, significance = 0.1%) during both the high and low rainfall periods. DistLM and ordination analyses identified temperature, total N, Si and salinity as significant variables that explained 36% of the variation in the eukaryotic community profiles, both spatially and between sampling periods (Fig 4). Temperature strongly correlated with community differences between the high (late summer) and low (early spring) rainfall periods, while salinity, total N and Si correlated with the spatial variations in the community.

For the prokaryotic phytoplankton mainly represented by *Synechococcus*, the clear distinction of community composition between the high and low rainfall periods strongly correlated with temperature, total N and PO_4_, with temperature alone explaining 85.6% of the variation (S6 Fig).

## Discussion

Little is known about the influence of periodic perturbations such as heavy rainfall on the primary producers that underpin the ecological functioning of a socially and economically important estuary, ‘rarely matched’ for habitat and biological diversity, the Sydney Harbour estuary [13]. By evaluating the spatio-temporal variability in the phytoplankton community during both high and low rainfall periods, we demonstrate how estuarine phytoplankton communities respond to stormwater inflow and discuss the potential implications for ecosystem functioning in the highly-biodiverse, yet impacted Sydney Harbour estuary.

### Variability in the phytoplankton community

Previous studies have shown that stormwater inflow into the Sydney Harbour estuary modifies the resident bacterial communities [28], with these shifts mainly considered within the context of a negative perturbation event, with implications for human health due to increased numbers of pathogens such as faecal coliforms [25]. Our data however, which focuses on the primary producers within this estuary suggests that a diverse and “healthy” phytoplankton community occurs within Sydney Harbour during the late summer high rainfall period. Specifically, the observed increase in total Chl-*a* together with the greater proportion of 16S rRNA sequences assigned to autotrophs suggests that overall phytoplankton abundance was likely higher than during the low rainfall period in early spring. This observation would appear to support previous findings of lower CO_2_ emissions from Sydney Harbour estuary during a high rainfall period, presumably indicating greater photosynthetic drawdown of CO_2_ [46]. This previous study [46] further demonstrated that during early spring, CO_2_ emission was higher, consistent with a switch towards net heterotrophic, rather than autotrophic status.

Furthermore, in addition to a likely higher overall abundance of phytoplankton during the high rainfall period, our study also found evidence of a phytoplankton composition conducive to a high-quality food web. Specifically, the apparent high abundance of diatoms (inferred from 18S rRNA gene), an important and highly-productive functional group, which typically support higher total biomass in upper trophic levels [6, 7], and are thus considered an essential part of estuarine ecosystem functioning [1]. Additionally, we observed no obvious indicators of ecosystem stress (e.g. hypoxia) commonly associated with localised eutrophication [47], despite exceptionally high nutrient concentrations close to stormwater point sources during the high rainfall period. Thus, from the perspective of studying photosynthetic microbes we found no evidence of negative ecological impact, rather, it appears that such transient perturbations may be beneficial for primary production.

In fact, during the low rainfall period, the relative phytoplankton abundance (inferred from 16S rRNA gene) and total Chl-*a* concentration were notably lower and the phytoplankton (based on 18S rRNA gene) was mostly represented by chlorophytes and dinoflagellates. While these findings should be interpreted with caution due to certain eukaryotes exhibiting multiple copies of the 18S rRNA gene which limits absolute quantification and abundance comparisons between phytoplankton classes [44, 45]; the substantial difference in the proportion of phytoplankton sequences likely reflects the difference in their abundance between the sampling periods.

### Environmental influence on phytoplankton composition

The apparent higher proportion of phytoplankton during the high rainfall period suggests environmental conditions conducive to phytoplankton growth. Temperature, salinity, total N and Si explained a third of the observed variation in phytoplankton community structure between high (late summer) and low (early spring) rainfall periods.

Temperature and light, are important factors regulating photosynthesis [4], and thus potentially phytoplankton growth rates. Temperature, which was considerably (6°C) higher in the late summer high rainfall period than the low rainfall period in early spring, certainly contributed to the difference in phytoplankton composition. Notably, this seasonally-driven difference in temperature was the most significant driving factor behind shifts in *Synechococcus* clade composition, which is consistent with patterns reported in other estuarine environments (e.g. Chesapeake Bay, see [48, 49]). The predominance of *Synechococcus* clade II in late summer (high rainfall period) and prevalence of clades I and IV in early spring (low rainfall period) is in accordance with clade-specific temperature niches, i.e. 20–28°C for clade II and 10–20°C for clades I and IV [50–53]. Although the seasonal difference in temperature partly explained differences in phytoplankton composition, numerous phytoplankton species, particularly diatoms which are a highly diverse group, flourish at a range of temperatures [1], therefore, it is highly likely that there are other factors also at play. Unfortunately, irradiance was not measured and therefore it remains unclear whether this had a significant influence on the observed differences in phytoplankton composition.

Changes in salinity and nutrients (N and Si) may be directly linked to stormwater inflow, and we demonstrate that in the high rainfall period, sites close to stormwater point sources exhibit markedly different phytoplankton communities with higher proportion of chloroplast to cyanobacterial 16S rRNA sequences, and the prevalence of diatoms based on 18S rRNA gene. Notably, coastal diatoms have a well-documented capacity to respond rapidly to macronutrient enrichment compared to other phytoplankton groups [54, 55]. Therefore, periodic delivery of N and Si via stormwater inflow may be an important driver of phytoplankton productivity within Sydney Harbour, which otherwise has limited nutrient enrichment from riverine input unlike most other estuaries [23]. This represents a potentially important finding particularly considering the recent prolonged drought that has affected the Sydney Harbour catchment area [56].

Finally, biotic interactions with predators such as grazers and/or viruses may also explain apparent lower phytoplankton abundance during the low rainfall period compared to the high raninfall period. Indeed support for this hypothesis is evident in the higher prevalence of zooplankton such as *Eucyclops* and *Leptodiaptomus*, known grazers of phytoplankton such as diatoms [57, 58], at sites with the lowest relative abundance of diatoms.

## Conclusions

Our data suggests that the community balance between heterotrophs and phototrophs is highly variable over space and time in the estuary, with a greater proportion and diversity amongst the phototrophic community during the high rainfall period, Therefore, by inference, such transient perturbations may in fact be beneficial for promoting biodiversity within Sydney Harbour. While we saw no negative consequences of eutrophication during this study, whether this observation would hold true in the longer term and whether stormwater inputs represent a net benefit versus detriment remain unclear. Critically, the inevitable trade-off between the spatial resolution that was necessary within such a large and spatially-heterogeneous estuary, means that we lack sufficient temporal resolution to capture potential phytoplankton successional dynamics in response to stormwater input. Since the last major assessment of the phytoplankton assemblages within Sydney Harbour estuary was conducted four decades ago [12], we have no reliable community baseline from which to evaluate the interesting findings of this study. However, by unravelling the phytoplankton diversity and the drivers of community change, our study provides insights into phytoplankton dynamics within this highly urbanised ecosystem. Further high-resolution monitoring to capture the interplay between eutrophication and phytoplankton productivity will be critical to understand how transient stress affects primary production, and help inform future management policy of the urbanised catchment.

## Supporting information

S1 Fig Physicochemical parameters of water from sites sampled within Sydney Harbour estuary during the high and low rainfall periods. **a)** Temperature (°C), **b)** Salinity (psu), **c)** pH (unitless), **d)** Total Suspended Solids (TSS, mg.L^-1^) and **e)** Chlorophyll (μg.L^-1^).
(TIF)

S2 Fig Nutrient concentrations of water from sites sampled within Sydney Harbour estuary, during the high and low rainfall periods. **a)** Nitrate/Nitrite (μg.L^-1^), **b)** Ammonium (μg.L^-1^), **c)** Total nitrogen (N, μg.L^-1^), **d)** Phosphate (μg.L^-1^) and **e)** Silicate (μg.L^-1^).
(TIF)

S3 Fig Multi-dimensional scaling (MDS) plot of the planktonic prokaryotic community profile, based on 16S rRNA gene (V6-V8 regions), along the Sydney Harbour estuary during high and low rainfall. Samples are colour-coded based on clusters (assigned using hierarchical cluster analysis with SIMPROF test based on Bray Curtis similarity) from high rainfall period: **1** (olive green), **2** (purple), **3** (red), **4** (blue), **5** (brown); and from low rainfall period: **6** (green), **7** (coral), **8** (pink). Regions of the sample sites are represented by symbols: Parramatta River (asterisk), Lane Cove (circle), Western Central Harbour (inverted triangle), Eastern Central Harbour (triangle), Middle Harbour (square) and Marine/Harbour Heads (diamond).
(TIF)

S4 Fig *Synechococcus* community composition, based on *petB* gene, along the Sydney Harbour estuary during sampling. **a)** Multi-dimensional scaling (MDS) plot of the community in the high (red) and low (blue) rainfall periods. Symbols represent geographic region sampled: Parramatta River (asterisk), Lane Cove (circle), Western Central Harbour (inverted triangle), Eastern Central Harbour (triangle), Middle Harbour (square) and Marine/Harbour Heads (diamond). **b)** Relative abundance of *Synechococcus* lineages based on *petB* gene sequences detected during high and low rainfall periods.
(TIF)

S5 Fig Relative abundance profile of the planktonic eukaryotic community, at the genus level, at each sampling site in the Sydney Harbour estuary during the high and low rainfall periods. (TIF)

S6 Fig **Distance-based redundancy analysis (dbRDA) of *Synechococcus* community composition (determined using *petB* gene) for the Sydney Harbour estuary, under high (red) and low (blue) rainfall conditions**. Symbols represent geographic region sampled: Parramatta River (asterisk), Lane Cove (circle), Western Central Harbour (inverted triangle), Eastern Central Harbour (triangle), Middle Harbour (square) and Marine/Harbour Heads (diamond).
(TIF)

S1 Table Environmental parameters at sampling sites in the Sydney Harbour estuary, during high (February) and low (September) rainfall periods. (XLSX)
