# Supplementary figures and images for: Stormwater influences phytoplankton assemblages within the diverse, but impacted Sydney Harbour estuary
Source: PLoS One. 2018 Dec 26;13(12):e0209857. doi: 10.1371/journal.pone.0209857 (PMC6306231; doi:10.1371/journal.pone.0209857)

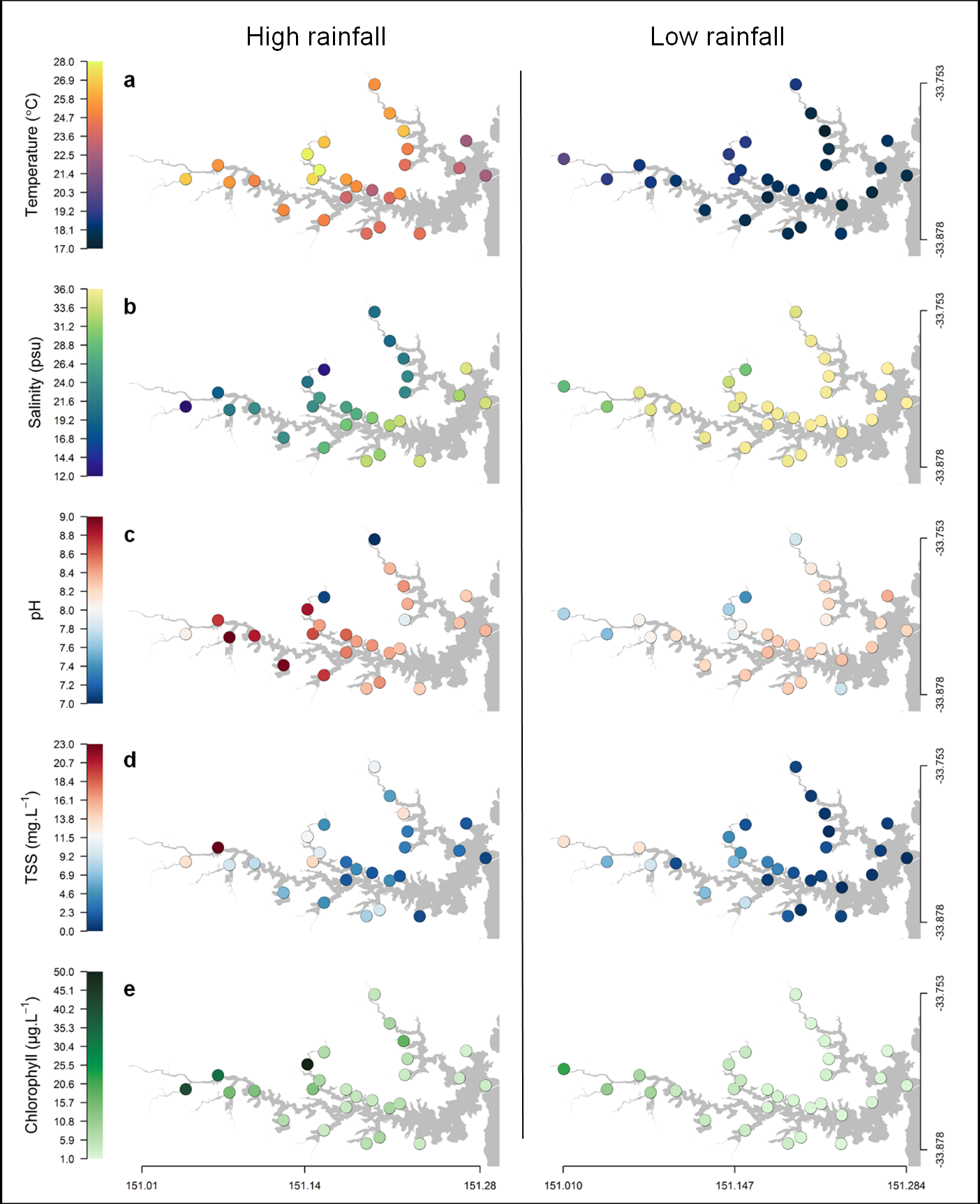

Supplement: S1 Fig — a) Temperature (°C), b) Salinity (psu), c) pH (unitless), d) Total Suspended Solids (TSS, mg.L-1) and e) Chlorophyll (μg.L-1). (TIF) [file pone.0209857.s001.tif]

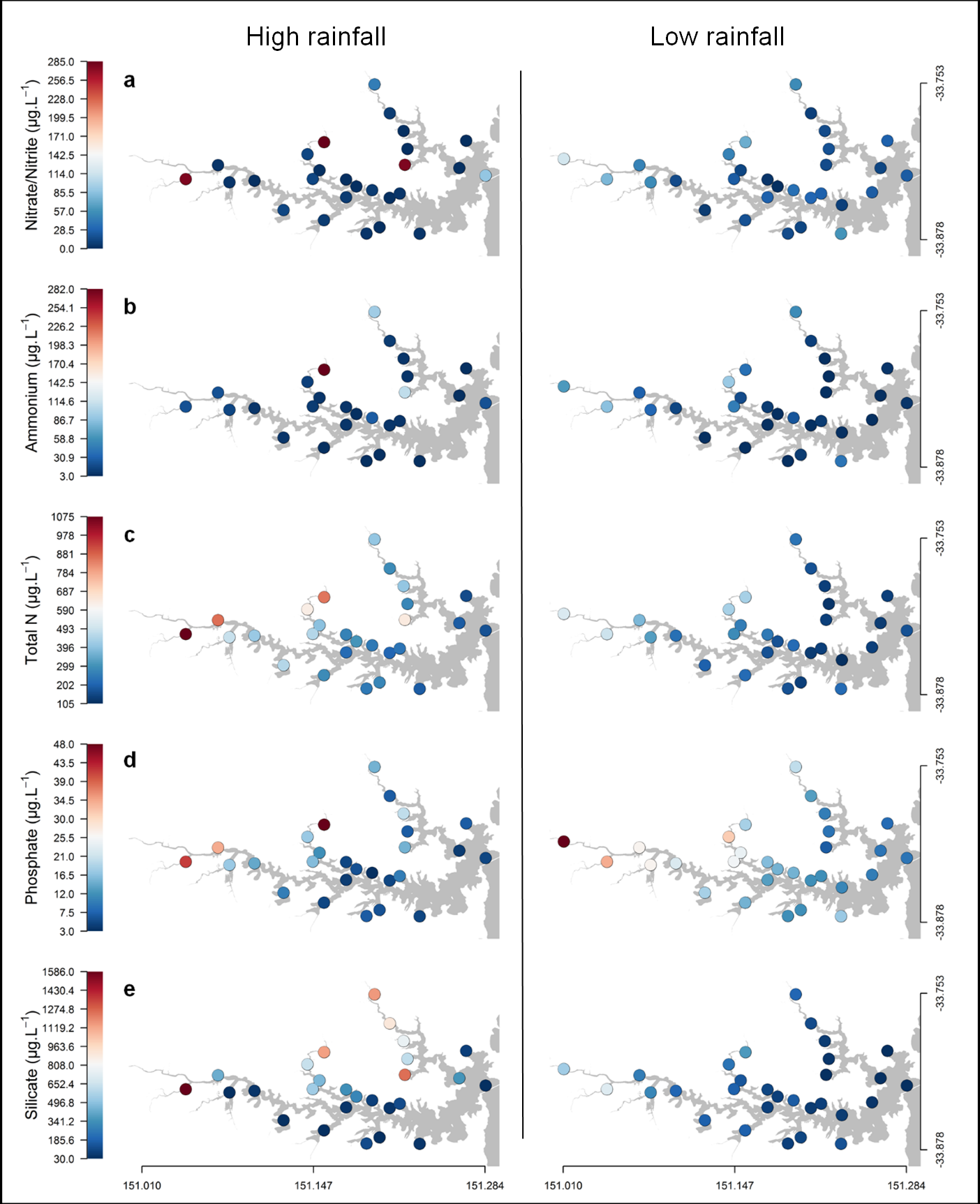

Supplement: S2 Fig — a) Nitrate/Nitrite (μg.L-1), b) Ammonium (μg.L-1), c) Total nitrogen (N, μg.L-1), d) Phosphate (μg.L-1) and e) Silicate (μg.L-1). (TIF) [file pone.0209857.s002.tif]

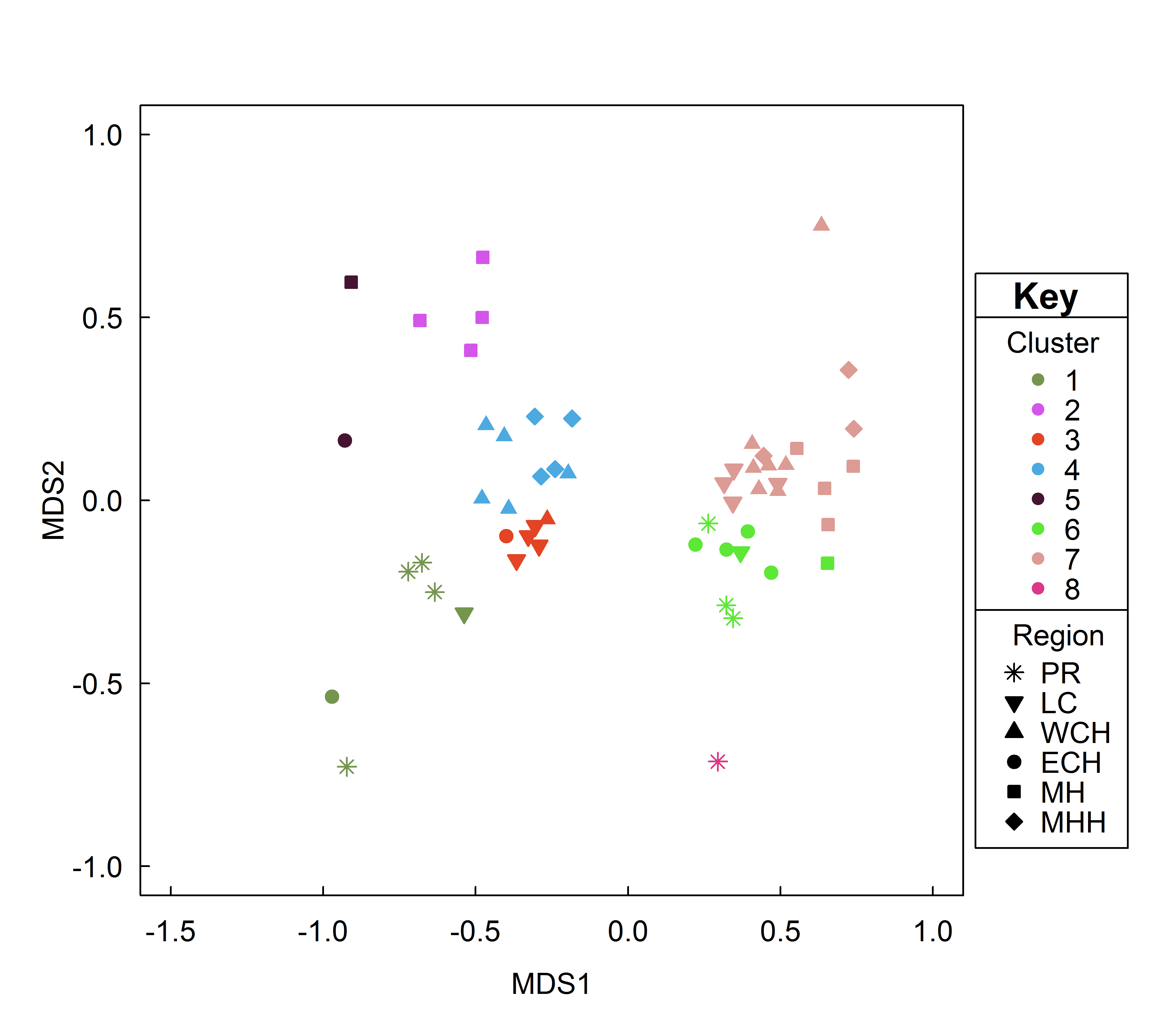

Supplement: S3 Fig — Samples are colour-coded based on clusters (assigned using hierarchical cluster analysis with SIMPROF test based on Bray Curtis similarity) from high rainfall period: 1 (olive green), 2 (purple), 3 (red), 4 (blue), 5 (brown); and from low rainfall period: 6 (green), 7 (coral), 8 (pink). Regions of the sample sites are represented by symbols: Parramatta River (asterisk), Lane Cove (circle), Western Central Harbour (inverted triangle), Eastern Central Harbour (triangle), Middle Harbour (square) and Marine/Harbour Heads (diamond). (TIF) [file pone.0209857.s003.tif]

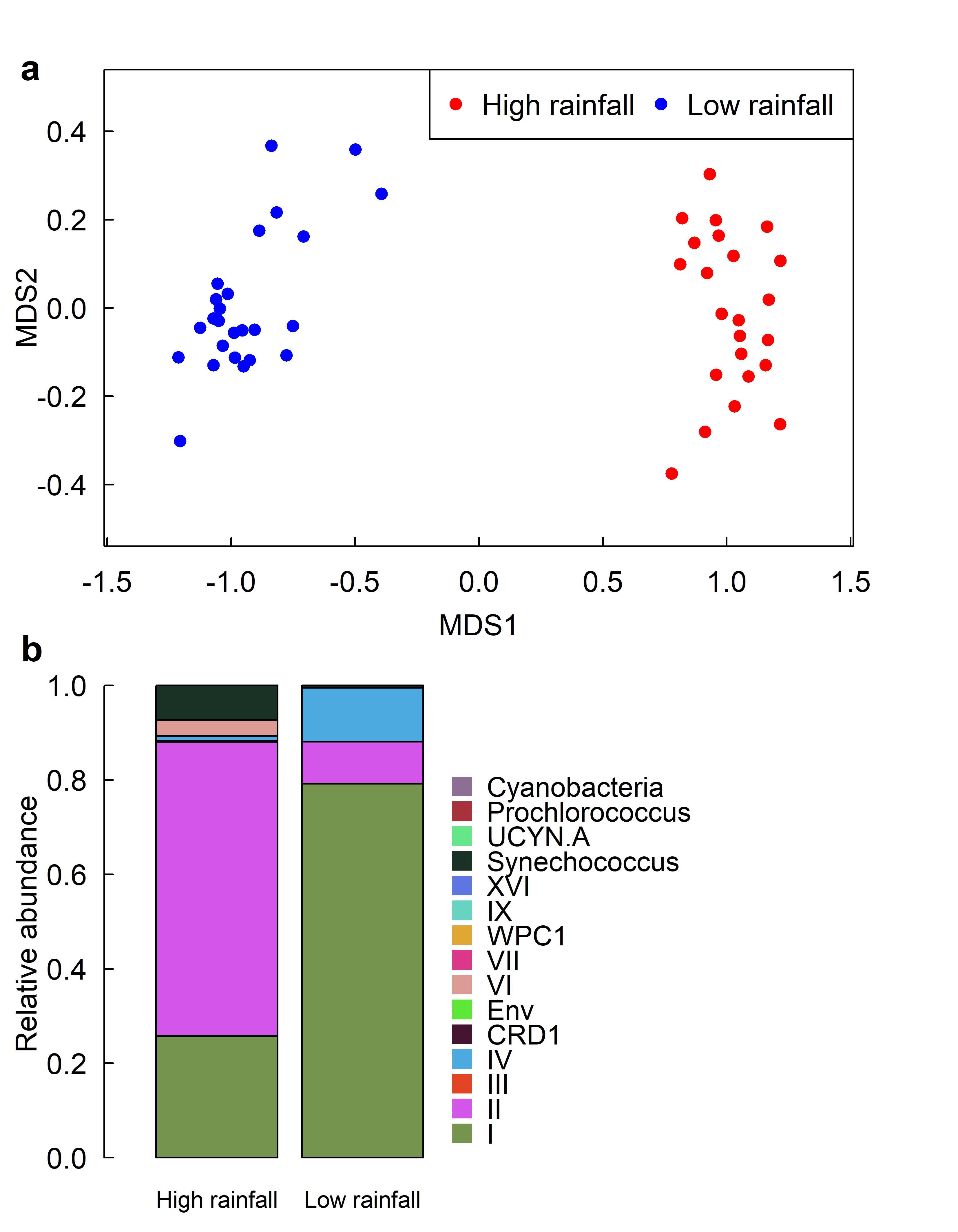

Supplement: S4 Fig — a) Multi-dimensional scaling (MDS) plot of the community in the high (red) and low (blue) rainfall periods. Symbols represent geographic region sampled: Parramatta River (asterisk), Lane Cove (circle), Western Central Harbour (inverted triangle), Eastern Central Harbour (triangle), Middle Harbour (square) and Marine/Harbour Heads (diamond). b) Relative abundance of Synechococcus lineages based on petB gene sequences detected during high and low rainfall periods. (TIF) [file pone.0209857.s004.tif]

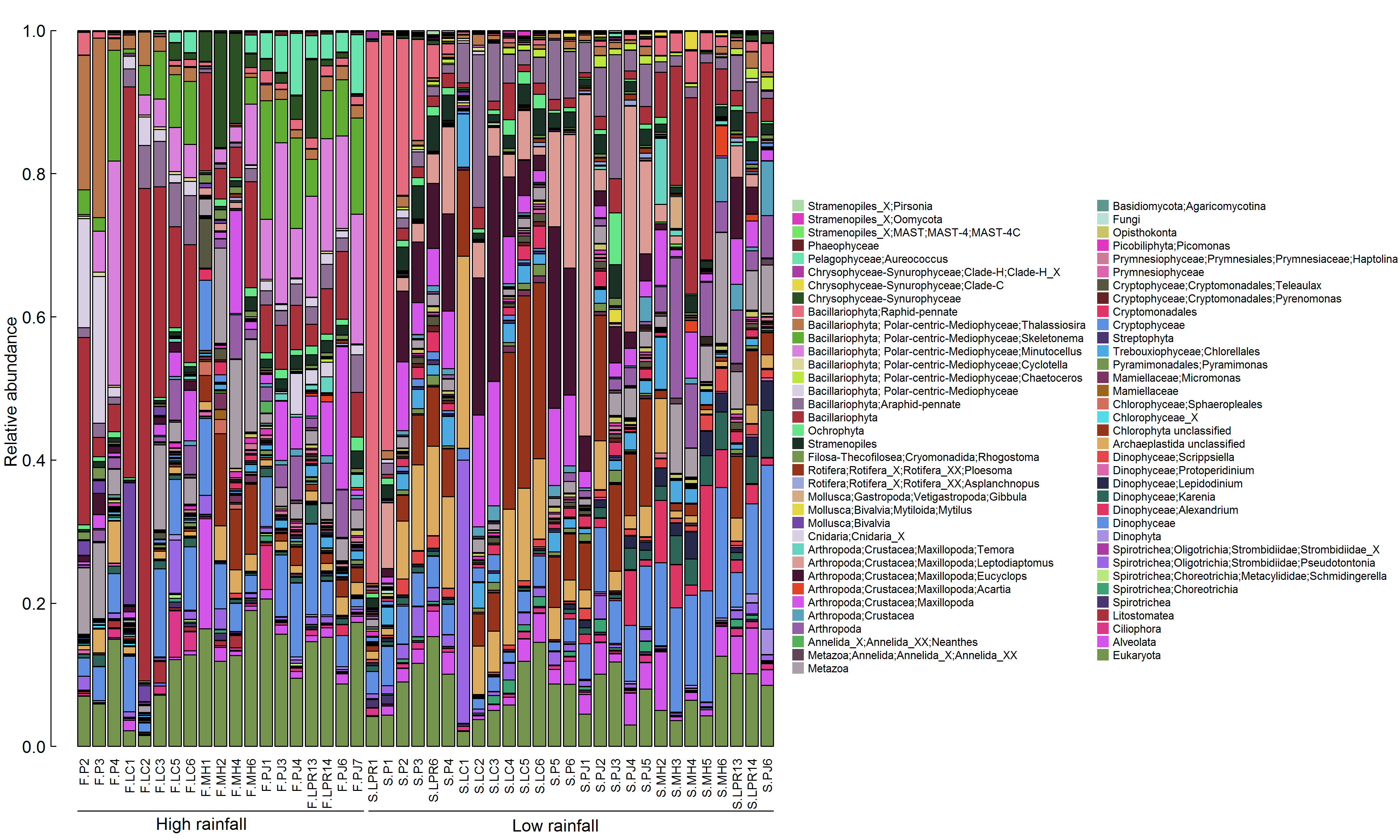

Supplement: S5 Fig — (TIF) [file pone.0209857.s005.tif]

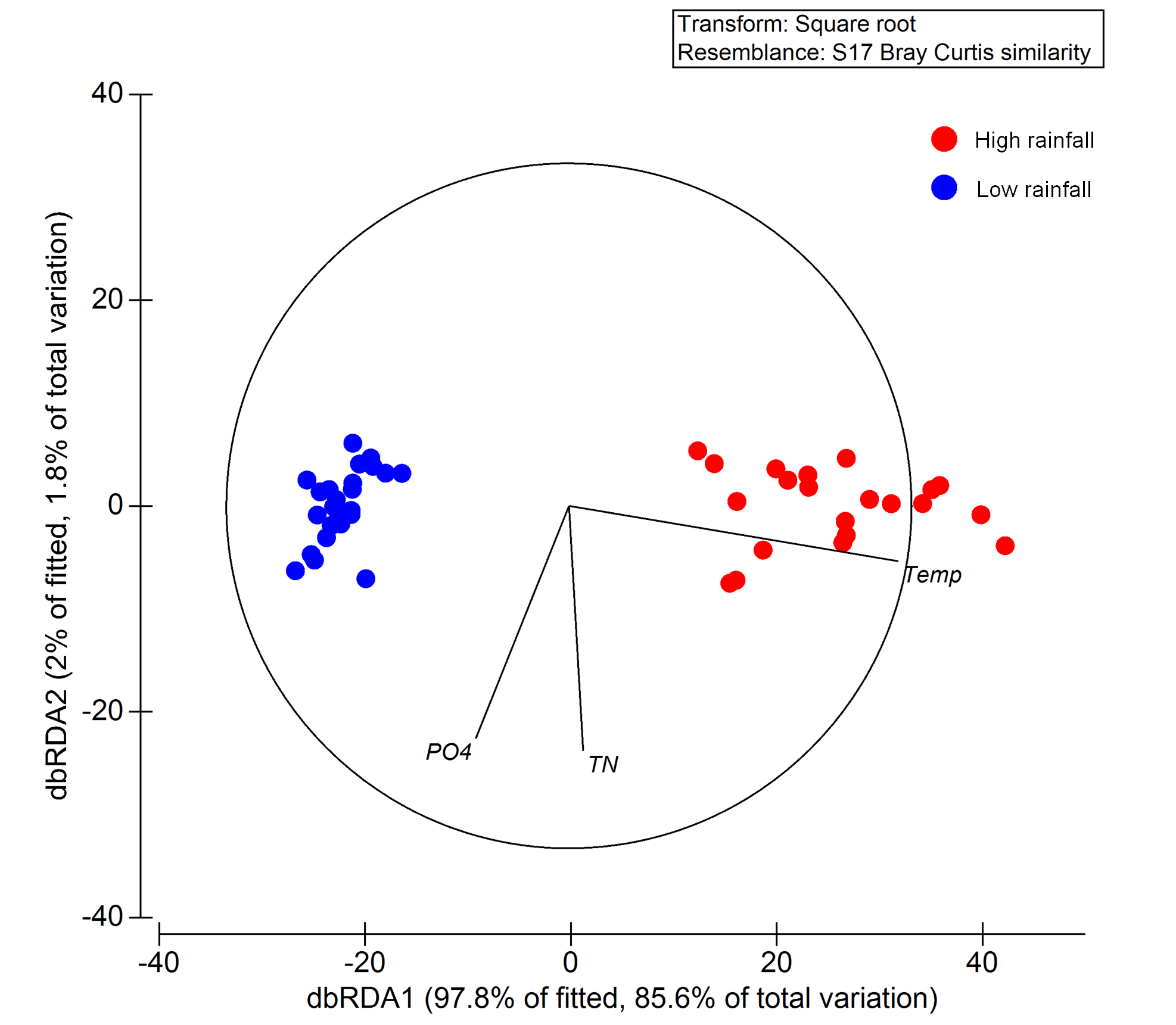

Supplement: S6 Fig — Distance-based redundancy analysis (dbRDA) of Synechococcus community composition (determined using petB gene) for the Sydney Harbour estuary, under high (red) and low (blue) rainfall conditions. Symbols represent geographic region sampled: Parramatta River (asterisk), Lane Cove (circle), Western Central Harbour (inverted triangle), Eastern Central Harbour (triangle), Middle Harbour (square) and Marine/Harbour Heads (diamond). (TIF) [file pone.0209857.s006.tif]
